# Using mathematical models to understand metabolism, genes, and disease

**DOI:** 10.1186/s12915-015-0189-2

**Published:** 2015-09-23

**Authors:** H. Frederik Nijhout, Janet A. Best, Michael C. Reed

**Affiliations:** Department of Biology, Duke University, Durham, NC 27708 USA; Department of Mathematics, Ohio State University, Columbus, OH 43210 USA; Department of Mathematics, Duke University, Durham, NC 27708 USA

## Abstract

Mathematical models are a useful tool for investigating a large number of questions in metabolism, genetics, and gene–environment interactions. A model based on the underlying biology and biochemistry is a platform for in silico biological experimentation that can reveal the causal chain of events that connect variation in one quantity to variation in another. We discuss how we construct such models, how we have used them to investigate homeostatic mechanisms, gene–environment interactions, and genotype–phenotype mapping, and how they can be used in precision and personalized medicine.

We began more than a dozen years ago creating mathematical models in order to understand the systems behavior of a variety of metabolic networks that are important for human health. In doing so, we were amazed at the plethora of regulatory and control mechanisms that have evolved to keep these systems functional in the face of genetic mutations and large changes in environmental inputs. The study of these regulatory mechanisms has led us, in turn, to devise methods to study four interlocking complex ideas: homeostatic plateaus, cryptic genetic variation, predisposition to disease, and precision or personalized medicine. The article is not intended as a review of the field, but as a description of our approach, the methods we have created, and our journey towards a practical understanding of these fundamental biological ideas.

A model gives voice to our assumptions about how something works. Every biological experiment is designed within the context of a conceptual model and its results cause us to confirm, reject, or alter that model. Conceptual models are always incomplete because biological systems are very complex and incompletely understood. Moreover, and as a purely practical matter, experiments tend to be guided by small conceptual models of only a very small part of a system, with the assumption (or hope) that the remaining details and context do not matter or can be adequately controlled.

Mathematical models are formal statements of conceptual models. Like conceptual models, they are typically incomplete and tend to simplify some details of the system. But what they do have, which experimental systems do not, is that they are completely explicit about what is in the model, and what is not. Having a completely defined system has the virtue of allowing one to test whether the assumptions and structure of the model are sufficient to explain the observed, or desired, results. This is one of the main points made by Jeremy Gunawardena in his essay that initiated this series of expository articles [1].

## Modeling is like experimentation

Mathematical models should not be ends in themselves. If they are to be of use, they should illuminate interesting things about the biology of a system or allow the user, by in silico experimentation, to discover things that would be difficult (e.g., severely reduce nutrient input), unethical (e.g., knock out or modify a gene in humans) or expensive (e.g., change the expression levels of different combinations of genes), or impractical to do in vivo or in vitro.

Ideally, a well-validated mathematical model is a tool, just like a microscope. It is a tool that complements other tools used in biological investigations, and it operates best (or at least most usefully) when there is an active interaction between modelers and experimenters. Experiments provide parameter values, functions and interactions that are essential for constructing the topology and kinetics of a model. A model, in turn, can suggest new experiments or help explain unexpected results. New experimental results improve a model and a model can guide the next round of experiments. Reciprocal illumination between the two should allow one to advance understanding more quickly and more accurately than would be possible with experimentation alone.

## Complex metabolic networks

We have focused most of our modeling studies on metabolic networks that are relevant to human health [2–13]. These systems are inherently interesting, and there are always large amounts of data and observations that need to be understood and explained. Sometimes data are contradictory or inconsistent, and that can lead to controversies; mathematical modeling can help provide explanations for the observed differences. More importantly, because these systems have been studied for a very long time there are a lot of structural and quantitative data that can be used to determine functional relationships and parameters. Metabolic systems are very complex and typically have many regulatory mechanisms (see below), so knowledge of the kinetics of individual reactions, though important, does not by itself explain the behavior of the whole system. In such a situation, mathematical models are essential to gain understanding at the systems level.

Since the purpose of our modeling is not to produce a model but to produce an exploratory and explanatory tool, we focus on systems where the kinetics of individual reactions or transporters have been well-studied experimentally. The aim of our work is to develop models that can help explain puzzling, conflicting, or contradictory experimental or clinical findings, and that can also be used to deduce the causal chain between genetic variation and phenotypic variation.

## Differential equation models and reaction kinetics

We normally work on metabolic systems in which the number of molecules of the species of interest is large enough that we can model the system by ordinary differential equations (ODEs) for the concentrations of the species (the well-mixed assumption). These differential equations simply reflect mass balance. The rate of change of a metabolite is the sum of the rates of the reactions by which it is made minus the rates of the reactions in which it is used. These rates are complicated, usually highly non-linear, formulas that express the rates as functions of the current values of one or more metabolite or allosteric regulator concentrations. These functions, the reaction kinetics, and the associated parameter values are obtained from the literature and from a large network of collaborators who perform experimental or clinical studies. The time courses of solution curves for different choices of inputs or parameter values are obtained by solving the ODEs in MatLab (Mathworks, Natick, MA, USA). If a system is large, we start by making mathematical models of small subsystems. Only when the subsystems are well understood do we study the whole system.

## Model testing

Models are inevitably limited, and can in fact be biased, by the data used to derive the equations and parameter values. This is especially problematic when a model is based on a small amount of data. It is essential, therefore, to continually test a model against new data that did not go into its construction. If the model cannot reproduce the basic trends in the data, then one knows that some new biological or biochemical ideas need to be added. On the other hand, if the model performs well with the new data, one’s confidence is increased that the model represents physiological reality. In this sense, the ‘model’ is not a fixed object, but continually evolves through testing it against data and revising it accordingly.

Of necessity one has to make a choice about the parameter values in a deterministic mathematical model. They are initially selected from the literature, although those values often vary considerably from publication to publication. The values are then gradually refined by testing the model against a broad diversity of data from experimental perturbations, dose–response studies, effects of mutations, and clinical studies. A good model should eventually reproduce, with some accuracy, a broad diversity of experimental data without having to fiddle with the parameter values. The result is a kind of ‘average’ or ‘normal’ model. Of course it cannot be expected to give an accurate representation of individual variability. To do that, we create population models based on our deterministic models (see below).

## The importance of homeostatic mechanisms

Metabolic systems are subject to hourly, daily, and seasonal variation in input and demand. Moreover, individuals and populations have different genetic makeups that can affect the expression and activity of enzymes and transporters in metabolic networks. Yet there are critical reactions and metabolite levels that must be maintained in the face of this environmental and genetic variation, or that must be adjusted to meet temporary increases in demand without compromising other important reactions in the system.

All the metabolic systems that we have studied contain homeostatic mechanisms that accomplish this task. These mechanisms include product inhibition (as one might expect), but also substrate inhibition and allosteric regulation, both positive and negative, of enzymes by metabolites in distant parts of the network. We will illustrate these mechanisms through several specific examples.

Substrate inhibition is an interesting phenomenon: as the concentration of substrate increases the rate of the reaction decreases because the substrate binds at allosteric sites that reduce the activity of the enzyme that uses it [14]. In the folate cycle, for instance, many enzymes are subject to substrate inhibition [3, 15–17]. As a consequence much of each enzyme is catalytically inactive under normal folate concentrations. When folate levels decline, some of the inactive enzyme–folate complexes dissociate, releasing new active enzyme and folate. Modeling has shown that in the absence of substrate inhibition flux through the pathway declines linearly with folate concentration, but in the presence of substrate inhibition reaction rates stay nearly constant until folate drops to less than 20 % of its normal level [3]. Folate substrate inhibition probably evolved as a buffering mechanism against seasonal variation in folate availability from fresh leafy greens in order to maintain critical reactions in the folate cycle, such as early steps in the synthesis of purines and pyrimidines for DNA synthesis. Substrate inhibition was emphasized by Haldane [18] in 1930, but it has been treated as a chemical curiosity in much of the literature even though some 20 % of all known enzymes show substrate inhibition [19]. In all cases that we have examined, substrate inhibition serves a biological purpose [14].

**Fig. 1:**
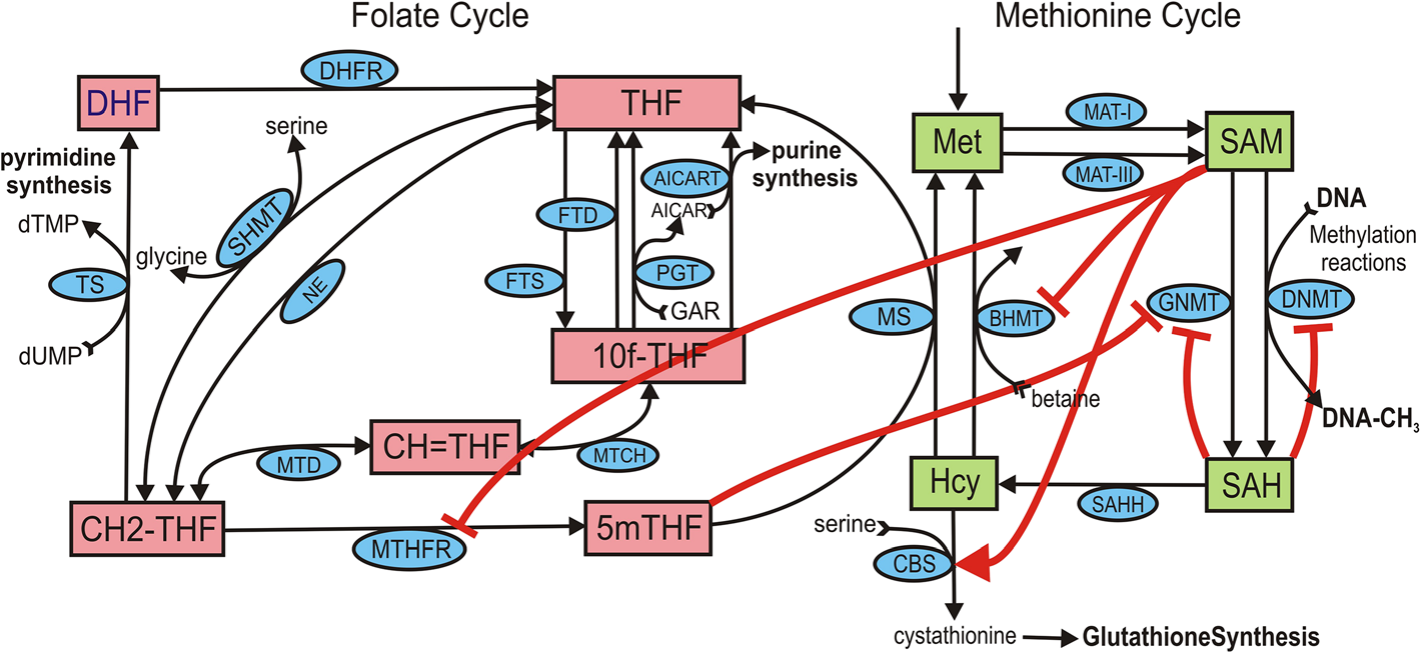
Topology of the folate and methionine cycles illustrating selected long-range allosteric regulatory interactions. Enzymes are indicated by *ellipses*, substrates by *rectangles*, and the allosteric regulatory actions by *red arrows*. These allosteric interactions serve to stabilize the DNA methylation reaction as follows. When S-adenosylmethionine (SAM) rises, due to increased methionine input, for instance, it inhibits 5,10-methylenetetrahydrofolate reductase (MTHFR). This reduces the level of 5-methyltetrahydrofolate (5mTHF), the co-substrate for methionine synthase (MS). This effect, together with the inhibition of betaine-homocysteine methyltransferase (BHMT), leads to a reduction in methionine synthesis. SAM also increases the rate of the CBS reaction, which removes the excess mass from the system. The reduction in 5mTHF, in turn, relieves inhibition of GNMT, causing more flux through this enzyme, which stabilizes the flux through the DNMT reaction until the level of SAM falls back to normal. Enzymes: *AICART* aminoimidazolecarboxamide ribonucleotide transferase, *BHMT* betaine-homocysteine methyltransferase, *CBS* cystathionine β-synthase, *DHFR* dihydrofolate reductase, *DNMT* DNA-methyltransferase, *FTD* 10-formyltetrahydrofolate dehydrogenase, *FTS* 10-formyltetrahydrofolate synthase, *GNMT* glycine N-methyltransferase, *MAT-I* methionine adenosyl transferase I, *MAT-III* methionine adenosyl transferase III, *MS* methionine synthase, *MTCH* 5,10-methenyltetrahydrofolate cyclohydrolase, *MTD* 5,10-methylenetetrahydrofolate dehydrogenase, *MTHFR* 5,10-methylenetetrahydrofolate reductase, *NE* non-enzymatic conversion, *PGT* phosphoribosyl glycinamidetransformalase, *SAAH* S-adenosylhomocysteine hydrolase, *SHMT* serinehydroxymethyltransferase, *TS* thymidylate synthase. Metabolites: *10f-THF* 10-formyltetrahydrofolate, *5mTHF* 5-methyltetrahydrofolate, *CH = THF* 5-10-methenyltetrahydrofolate, *CH2-THF* 5-10-methylenetetrahydrofolate, *DHF* dihydrofolate, *Hcy* homocysteine, *MET* methionine, *SAH* S-adenosylhomocysteine, *SAM* S-adenosylmethionine, *THF* tetrahydrofolate

Allosteric regulation of enzymes has equally significant consequences. As an example, we take the case in Fig. 1 that shows the methionine and folate cycles with four allosteric regulations indicated by the red arrows. S-adenosylmethionine (SAM) is the universal methyl donor in cells, in particular for DNA-methyltransferase (DNMT), the reaction that methylates DNA. The level of SAM rises and falls with methionine input. When SAM starts to fall the inhibition of 5,10-methylenetetrahydrofolate reductase (MTHFR) is released, resulting in more 5-methyltetrahydrofolate (5mTHF), which in turn drives the methionine synthase (MS) reaction and inhibits the glycine N-methyltransferase (GNMT) reaction. Thus, more methionine is resynthesized and fewer methyl groups are lost through the GNMT reaction. The reverse sequence of events happens if SAM starts to go up. The result is that the level of SAM does not change very much and the rate of the DNMT reaction stays fairly constant. This wonderful mechanism was first elucidated by Wagner and co-workers [20]; we showed quantitatively how stable the DNMT reaction rate is to stochastic variation in methionine input [2].

A quite different homeostatic mechanism occurs in the stabilization of dopamine (DA) in synapses. The concentration of DA in the extracellular space is maintained by a balance between release from the terminal and reuptake by the DA transporters (DATs), and by autoreceptors that sense the DA concentration in the extracellular space [21, 22]. When the concentration goes down the autoreceptors increase the activity of tyrosine hydroxylase (TH), the first enzyme in the two-step conversion of tyrosine to dopamine [23], and the rate of release of DA from synaptic vesicles [24–26]. If extracellular DA is too high, the opposite occurs. We developed a mathematical model for DA synthesis and release [5, 27, 28], and showed that the autoreceptor mechanism is very effective at stabilizing extracellular DA.

**Fig. 2:**
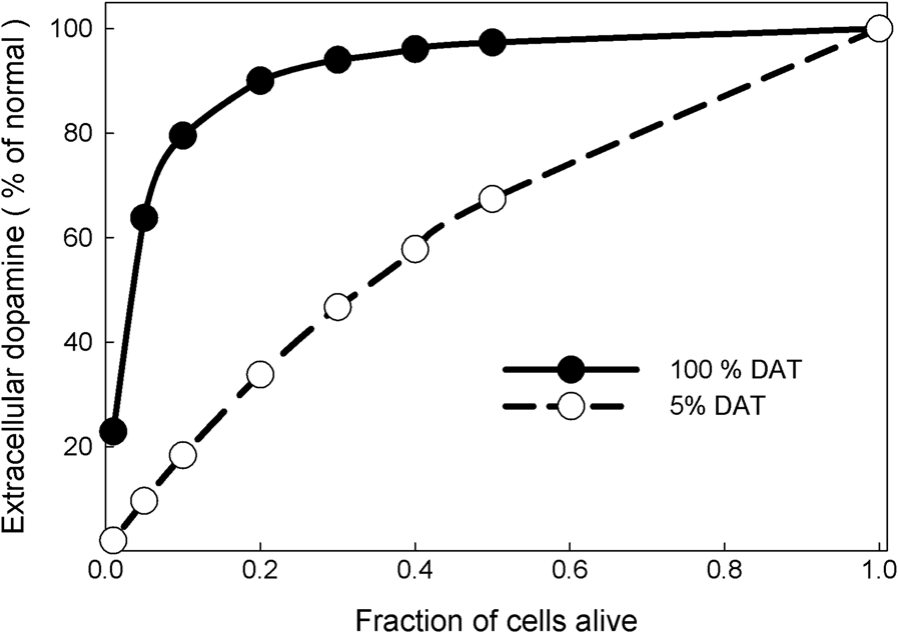
Homeostasis of extracellular dopamine. Model simulations of the effect of progressive cell death on the concentration of extracellular dopamine. *Black*: dopamine concentration declines less than 10 % until more than 80 % of cells have died. *White*: effect of reducing the activity of the dopamine reuptake transporter (DAT); now the level of extracellular dopamine is very sensitive to the size of the cell population. After [4, 26]

Decline of extracellular DA in the striatum, due to the death of DA neurons in the substantia nigra (SN), is the immediate cause of Parkinson’s disease (PD). Our mathematical model showed that the autoreceptors, together with the DATs, serve to stabilize extracellular DA in the face of massive cell death in the SN. Our models showed that as cells die extracellular DA drops slightly but does not begin to decline significantly until more than 80 % of the dopaminergic neurons have been lost (Fig. 2) [5]. This figure is in accord with clinical and postmortem findings that show that PD symptoms typically do not begin to appear until about 80 % of the neurons in the SN have died.

## Dynamic stability against short-term variation

**Fig. 3:**
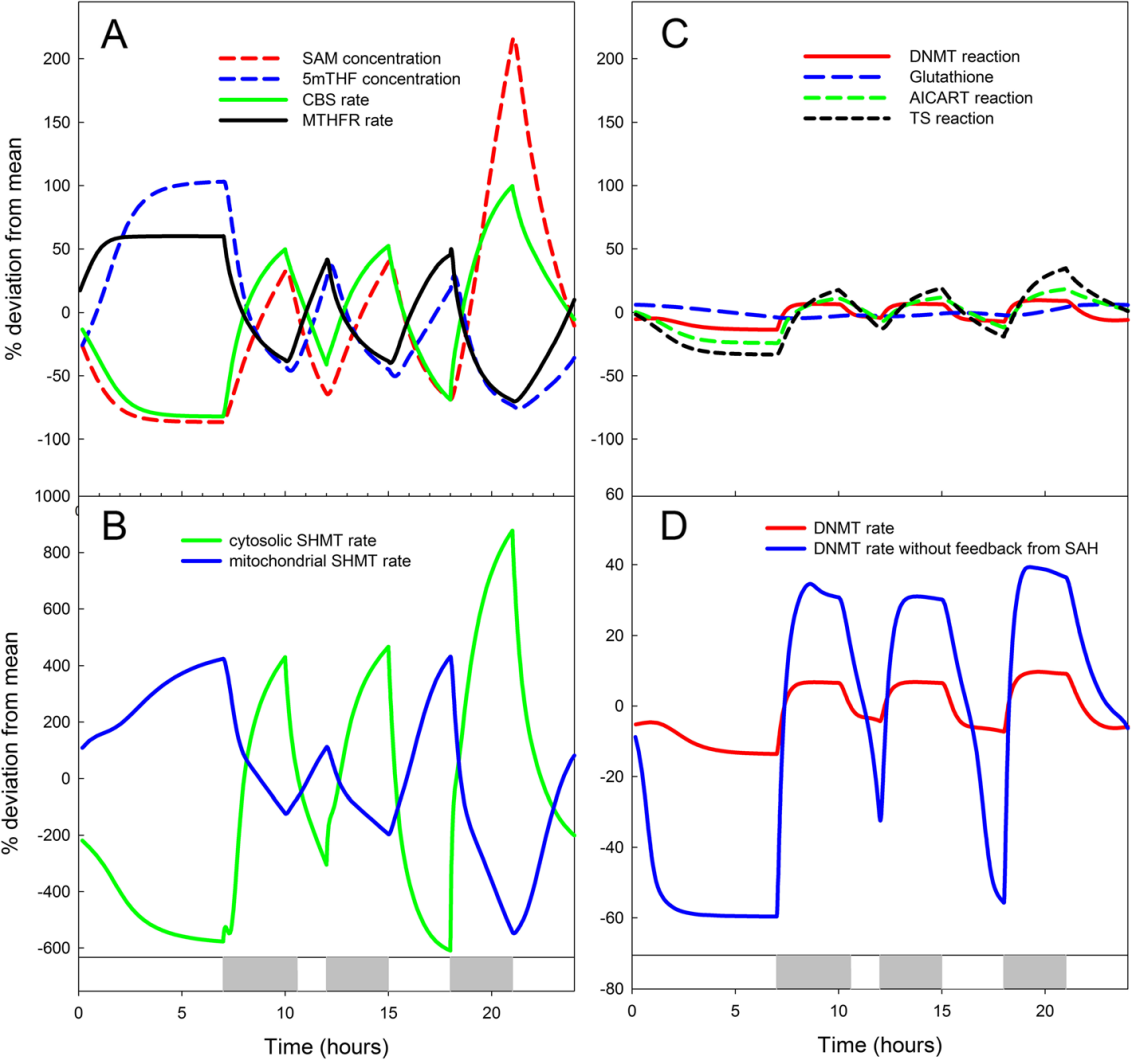
Effect of short-term variation in amino acid input on metabolite levels and reaction velocities in the folate and methionine cycles. Three pulses of amino acids are shown by *gray bars* below the figures, corresponding to three meals over a 24-hour period. Variation in response is shown as percentage deviation from the mean. **a** Two metabolites (SAM and 5mTHF) and reaction velocities [cystathionine β-synthase (*CBS*) and MTHFR] that show complementary responses. **b** Reaction velocities of mitochondrial and cytosolic serinehydroxymethyltransferase (*SHMT*; values below −100 % are reversals of direction of the reaction). **c** Dynamic variation of fluxes throughout the pathway stabilize the velocities of DNMT, TS, and AICART and the concentration of glutathione. **d** Eliminating product inhibition by S-adenosylhomocysteine (*SAH*) increases the sensitivity of the DNMT reaction to variation in input is indicated by the greater amplitude of the response. After [28, 35]

Short-term fluctuations in input are inevitable in metabolic systems because inputs of metabolites can change dramatically after each meal. The role of the homeostatic mechanisms in damping the effect of fluctuating inputs can be illustrated by varying the amino acid input terms in the model to reflect the changes in their blood values during and after meals over a 24-hour period [29]. In Fig. 3 we see the effect of three meals on a few concentrations and reaction velocities in an enlarged model of the folate-mediated one carbon metabolism (FOCM) system that also includes the mitochondria and the synthesis of glutathione (GSH) [4]. The fluxes in different parts of the system vary enormously with meals, except for three critical reactions (e.g., thymidylate synthase, the rate-limiting step for DNA synthesis, and DNA methyl transferase), and the concentration of GSH (Fig. 3c), which vary little if at all. This system has the property that many reactions and substrate levels change dramatically in order to maintain stability of a few critical ones. This is typical of physiological homeostasis. By removing the feedbacks one-by-one, or in different combinations, one can study the degree to which each contributes to the stabilization of each of the four critical reactions. For instance, we showed that the inhibition of MTHFR by SAM has the biggest effect on stabilizing the DNMT reaction, whereas the stimulation of cystathionine β-synthase (CBS) by SAM has a smaller effect [2]. The well-known mechanism of product inhibition also has important stabilization effects. S-adenosylhomocysteine (SAH) inhibits all the methyl transferases. Figure 3d shows how this product inhibition helps stabilize the DNMT reaction.

## Stabilization against genetic variation

**Fig. 4:**
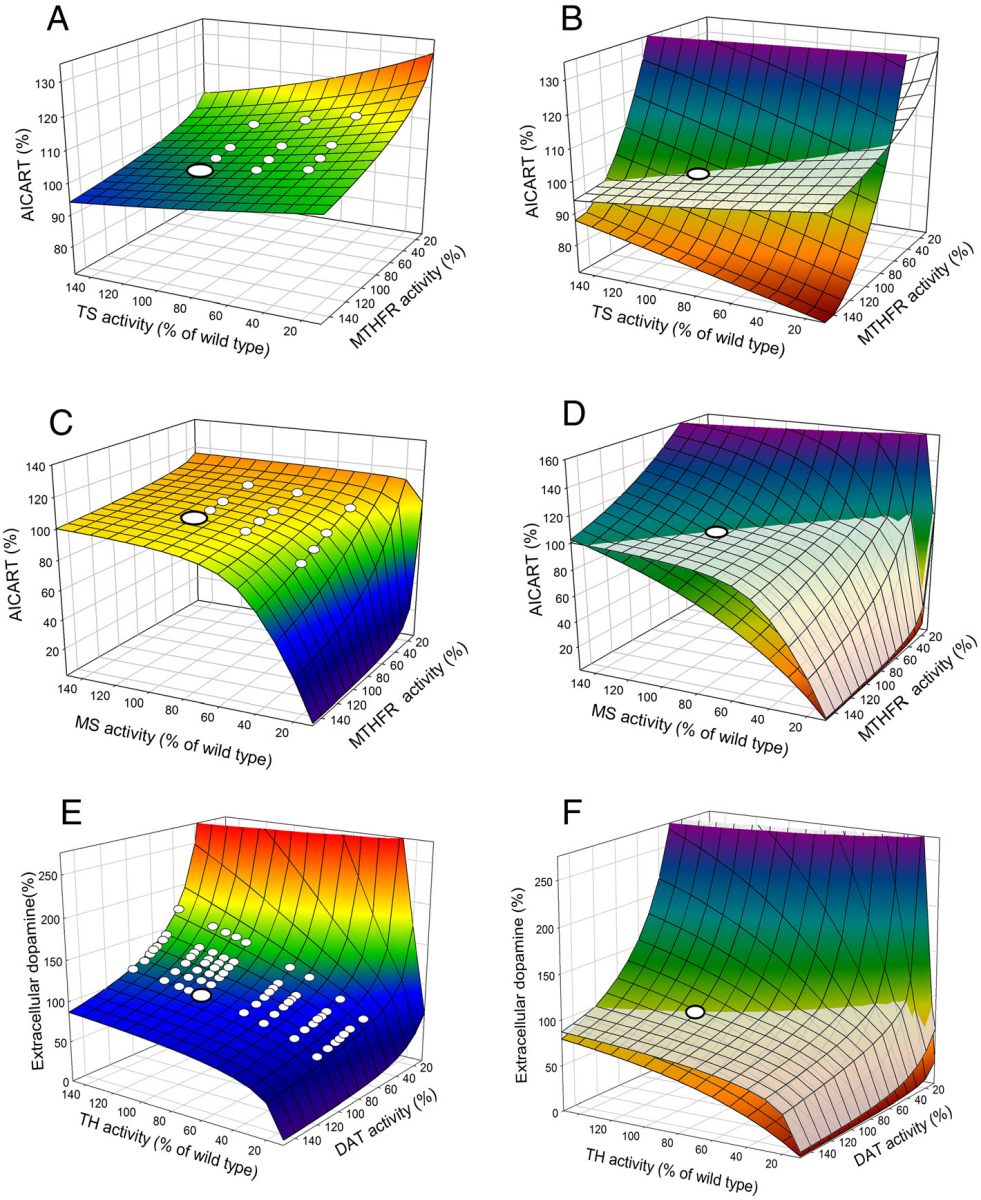
Robustness of phenotypes against genetic variation. These figures are phenotypic landscapes that illustrate the effects of pairwise combinations of ‘genetic’ variables (x and y axes) on selected phenotypes (z axis). The genetic variables are enzyme activities shown as percentage of wild type. The *large white circles* indicate the position of the wild type. The *small white circles* are the values for various mutations in the underlying genes (taken from Table 1). **a**, **c** Stability of the AICART reaction against genetic variation. The wild type and most mutations lie on a relatively flat horizontal portion of the phenotypic landscape. Thus, even mutations with large effect at the molecular level can have only a minor effect at the phenotypic level. **b**, **d** The effect of removing the inhibition of GNMT by 5mTHF on the shape of the phenotypic landscape. The grey landscapes are from the left panels and the colored landscapes show the effect of removing the feedback regulation. After [35]

Our models for FOCM and dopamine and serotonin [11, 30] metabolism have shown that these homeostatic mechanisms also stabilize critical phenotypes (the rates of DNA methylation and the thymidylate synthase reaction, and the concentration of synaptic DA, for instance) against genetic variation. Given that these metabolic systems are critical for human health, and that defects are strongly associated with a variety of disease states [31–35], we were surprised to find that many of the genes for enzymes in these metabolic systems have large-effect high-frequency polymorphisms in human populations, and the natural question arises as to why these defective genes persist. Table 1 shows a selection of polymorphisms, their effects on enzyme activity, and their frequency in selected populations. It turns out that although the effects of these mutations are quite large at the molecular level, the homeostatic mechanisms greatly reduce their effect at the phenotypic level. This can be illustrated by a phenotypic landscape graph in which we plot the phenotype as a function of simultaneous variation in two of the enzymes (or transporters) in the system [36, 37]. Two such landscapes are illustrated in Fig. 4. As can be seen in Fig. 4a,c, the ‘normal’ genotype, which we’ll call the wild type, lies in a region where the landscape is quite ‘flat’ (or, rather, orthogonal to the phenotypic axis). This means that genetic variation around the wild type will have little or no effect on the phenotype. In fact, the polymorphisms from Table 1 are almost all on the flat regions of the surfaces. This illustrates that big effects at the enzymatic level (x and y axes) can have little effect at the phenotypic level (z axis). Thus, these polymorphisms constitute what evolutionary biologists call cryptic genetic variation. Fig. 4b,d show what happens when one of the regulatory mechanisms (the inhibition of GNMT by 5mTHF) is removed. The landscapes change shape dramatically and are no longer orthogonal to the phenotypic axis. Thus, an additional mutation that destroys this regulation would uncover the accumulated cryptic genetic variation and cause it to become phenotypic.

**Table 1:** Common large-effect mutations in FOCM and dopamine metabolism, their effects on the activities of the respective enzymes, and frequencies in selected populations^a^

Gene/enzyme	Mutation	Activity relative to wild type	Gene frequencies^b^
MS	A2756G	50 %	9 % (C), 16 % (US), 20 % (EU)
MS	D919G	60 %	17 % (J), 55 % (US)
MTHFR	C677T	30 %	51 % (I), 34.5 % (ME), 35 % (US)
MTHFR	A1298C	68 %	33 % (I), 33 % (US)
TS	2rpt/3rpt	42 %	48 % (US), 40 % (EU), 8 % (C)
TS	1494del6	24 %	76 % (US), 33 % (C)
CBS	M173V	38 %	-
CBS	A226T	13 %	4.5 % (AA)
CBS	R548Q	60 %	0.6 % (S)
CBS	T191M	10 %	14–75 % (H)
TH	T245P	150 %	-
TH	T283M	24 %	-
TH	T463M	116 %	-
TH	Q381K	15 %	Familial
DAT	V382A	48 %	
DAT	hNET	65 %	
DAT	VNTR10	75 %	

^a^For references see [34,35]

^b^ *US* United States, *EU* Europe, *C* China, *J* Japan, *I* Italy, *S* Spain, *AA* African Americans, *H* Hispanics, *ME* Middle East

*CBS* cystathionine β-synthase, *DAT* dopamine reuptake transporter, *MS* methionine synthase, *MTHFR* 5,10-methylenetetrahydrofolate reductase, *TH* tyrosine hydroxylase; *TS* thymidylate synthase

## Predisposition to disease

**Fig. 5:**
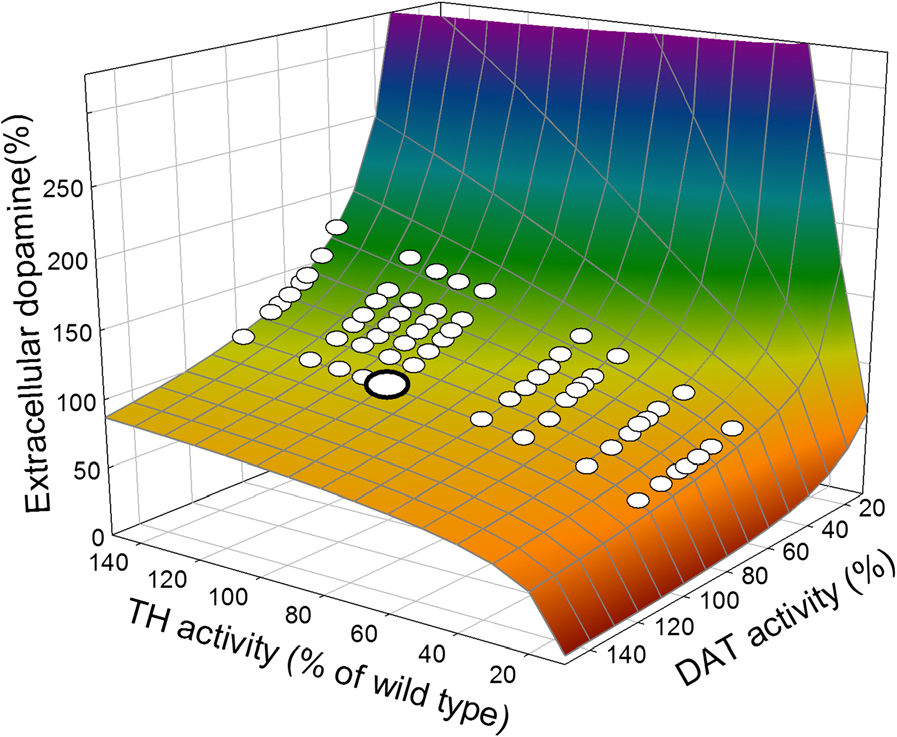
Stability of extracellular dopamine against genetic variation. Homeostasis of dopamine to variation in the activities of TH and DAT. The wild type is indicated by the *large white circle*. The positions of homozygotes and heterozygotes for the seven mutations from Table 1 are indicated by *small white circles*. The alleles are assumed to act additively. Most of the mutations lie in the relatively flat region of the landscape. After [26, 34]

The etiologies of some diseases are well understood, but in many cases the disease is a collection of symptoms that we have named. Fig. 4c shows aminoimidazolecarboxamide ribonucleotide transferase (AICART) activity as a function of methionine synthase (MS) activity and MTHFR activity. Very low AICART activity would likely be detrimental as cells would have difficulty dividing because their de novo synthesis of nucleotides would be impaired. The genotypes with only 30 % activity of MS are very close to the edge of the cliff over which the AICART activity declines precipitously. It is tempting to consider such individuals to be ‘predisposed’ to an AICART-dependent disease because a change in some variable not pictured (another mutation or an environmental factor) could push them over the cliff. Similarly, consider the surface in Fig. 5 where the phenotypic variable is the extracellular DA concentration and the genetic variables are the activity of TH and the DAT. The genotypes with very low TH activity are at the edge of the DA cliff and, interestingly, these genotypes sometimes show a dystonia [38], involuntary muscle contractions that affect posture, brought about by low levels of extracellular DA that can be alleviated by levodopa. Their placement at the edge of the cliff predisposes them to the dystonia, which can be brought on by changes in the other variables that affect the shape of the surface.

## Personalized medicine

We have shown only a few genotype–phenotype surfaces, but, of course, we can create such surfaces for all the genotypic variables (enzyme and transporter activities) and phenotypic variables (concentrations, reaction velocities) in our models and place an individual who has been genotyped at a particular spot on each of the surfaces. If the individual is near the edge of a cliff, medical advice could suggest lifestyle changes (diet) or drug therapy that would make the region around the genotypic point flatter and more homeostatic. We give two examples to explain how our mechanistic models could be used to do just that.

Suppose that an individual has very low MS activity and therefore very low AICART activity (Fig. 4c). To intervene wisely one has to know what the causal connection is between MS and AICART; it’s not obvious since they are not near each other in the network (Fig. 1). It’s easy to see in the model (simulations not shown) that when MS has very low activity then the concentration of 5mTHF gets very high and the other folates get very low (a situation know as the folate trap, because all folates are eventually converted to 5mTHF), so 10-formyltetrahydrofolate (10f-THF) declines and cannot drive the AICART reaction, which results in a diminished ability to synthesize nucleotides. Thus, the goal of the therapy is to reduce the folate trap. This could be done by giving folate, or it could be done by giving vitamin B12, which is a co-factor for the MS reaction. High homocysteine in the plasma is a biomarker for the folate trap, so in either case one can test the efficacy of the strategy by measuring the decrease in homocysteine.

As a second example, consider the genotypes that are close to the DA cliff in Fig. 5 because they have low TH activity and are therefore at risk for dystonia. A good therapeutic strategy should move them away from the edge of the cliff, or make the region around the defective genotype flatter. Our simulations (not shown) indicate that this can be done by increasing the strength of the DA autoreceptor effect, either by giving autoreceptor agonists or by increasing the expression level of the DA autoreceptors.

## Biological variation and virtual population models

What does biological variation mean? It means, for instance, that no two individuals have exactly the same folate metabolisms in their liver cells. There are many reasons for this. Their genotypes are different and that affects the activity of various folate enzymes. The expression levels of the genes that code for these enzymes vary in time depending on what the cell is doing, and are influenced by endocrine factors. Furthermore, during the day, enormous changes in amino acid inputs occur due to meals. Finally, many of the enzymes require vitamin co-factors, and so the reactions depend to some extent on individual dietary histories over months and years.

**Fig. 6:**
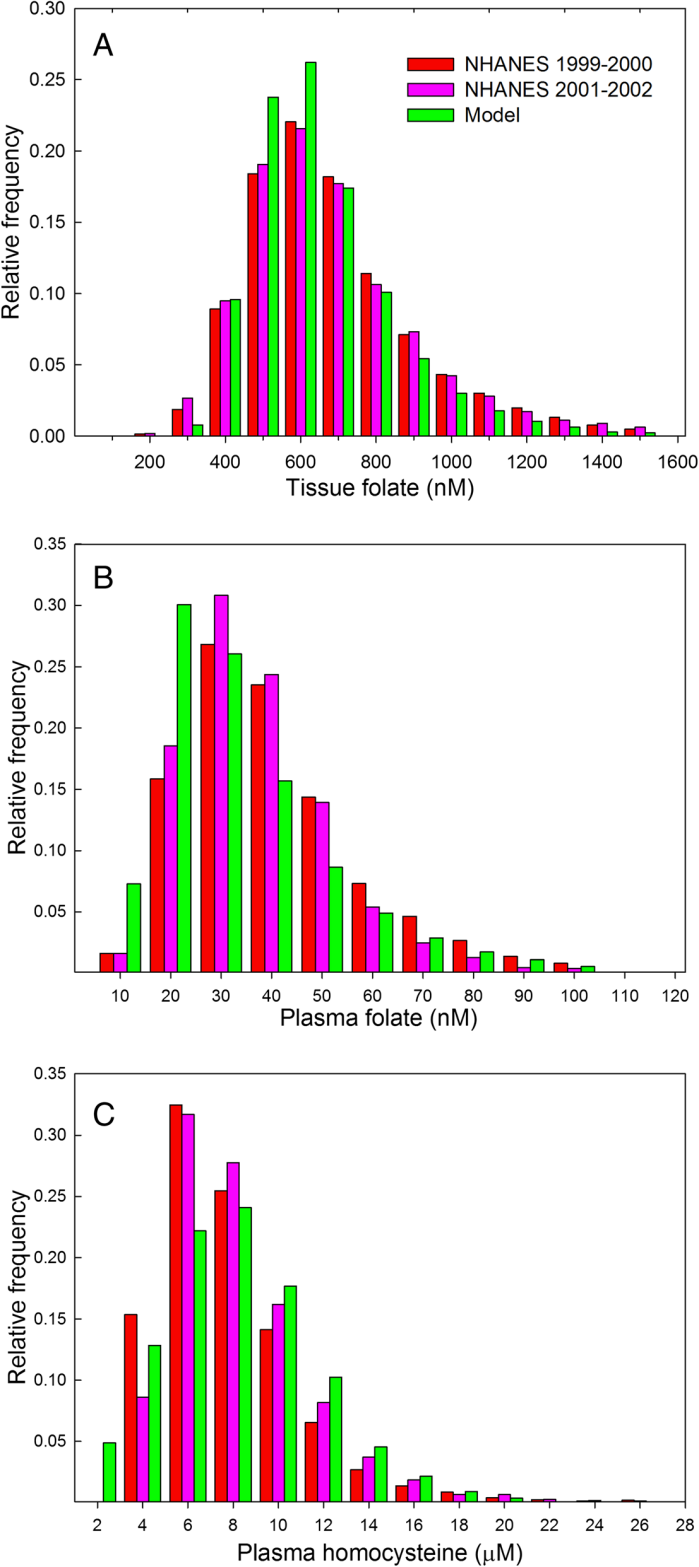
A population of virtual individuals. Adding random variation to the parameters of a deterministic model makes it possible to develop a population of virtual individuals, each with a unique combination of genetic and environmental parameters. Here we illustrate that the frequency-distribution of tissue folate (**a**), plasma folate (**b**), and plasma homocysteine (**c**) closely matches the corresponding data in two NHANES databases (modified from [12])

To represent individual variation and diversity, we make population versions of our models. A virtual individual is created by selecting each parameter from a distribution centered on its normal value, and then running the program to steady-state, and recording the concentrations and velocities, which represent the phenotypic values for that individual. If we repeat this process 10,000 times, we get a database of 10,000 virtual individuals that contains both genetic variation (in terms of variation in the V_max_ and K_m_ of enzymes), and environmental variation (in terms of variation in nutrient and micronutrient levels), and the resulting phenotypic values [12]. Comparison of the results of such a virtual population model with extant databases gives another way of verifying that the deterministic model represents physiological reality well. Fig. 6, for instance, compares the frequency distributions of tissue folate, plasma folate, and plasma homocysteine in a virtual population model [12] with the corresponding distributions in two National Health and Nutrition Examination Survey (NHANES) studies. As one can see, the fits are quite good.

Such a virtual population database can now be studied to discover interesting statistical relationships between phenotypic, genetic, and environmental variables. The population model is based on a deterministic model, so we can use the deterministic model to discover the causal chain of events that leads to the statistical relationships seen in the database. In this way, statistical analyses, which deduce the correlated variation among variables, and deterministic mathematical models, which can show how those variable are causally linked, can work together to elucidate underlying biological function.

## Limitations and alternative strategies

All methods and tools have limitations and that is true of our approach too. Since we stay very close to the underlying biology and biochemistry, we can only study metabolic systems where there is a great deal of information about the component parts and the kinetics of how they affect each other. Nevertheless, our approach can be used in other areas besides cell metabolism. There are some outstanding examples in physiology, such as the regulation of blood pressure [39], and kidney function [40, 41]. Other examples can be found in epidemiology [42, 43], immunology [44], cancer biology [45, 46], regulation of the cell cycle [47, 48], insulin and diabetes [49–51] and various examples worked out in [52].

For biological systems in which little is known about details or kinetics, phenomenological models that capture some aspect of the data may be helpful in clarifying ideas and suggesting targets for experimentation. For example, this is surely the case in studying the brain, where the chasm between experimentation at the neural level and behavior is so vast that models using hypothetical intermediate variables can be valuable. However, we remark again that experimentation with such models will tell us things about the underlying biology only if the parameters have biological meanings and could potentially be measured by experimentalists.

An approach, favored by some systems biologists, is to use correlations between phenotypic variables measured in a population (people or cells) to reverse engineer the network itself and then to assign strengths to the interactions between the variables by using machine learning and parameter estimation. We believe such methods are unlikely to succeed because the underlying biological processes are highly nonlinear, and nonlinear in many different ways, whereas statistical learning approaches to parameter estimation depend on linear models or on models that allow only a restricted type of nonlinearity. It is unlikely that such approaches could find the roles of allosteric activation, substrate inhibition, and dynamic stability to short-term perturbations, robustness to genetic variation, and gene–environment interactions that we see in biology, and in our models. Our virtual population databases could, of course, be used to test whether a reconstruction method can accurately deduce the structure and kinetics of the network that generated the data.

Statistical approaches on large data sets can be useful for revealing interesting correlations and relationships between phenotypic and genetic variables. As we indicated above, we can derive interesting and useful information by statistical analyses on our virtual population databases. And in systems where the biology is largely unknown, statistical methodology may be the only useful set of tools that one has. However, one must always remember that correlation is not causality. Until one understands mechanism, designing experiments or intervention strategies (whether via drugs, life style changes, or surgery) based on the model is difficult and risky.

## Conclusions

The main point of this article is to explain that mathematical models are a useful tool for investigating a large number of questions in metabolism, genetics, and gene–environment interactions. If the model is based on the underlying biology and biochemistry, then it becomes a platform for in silico biological experimentation and it can also reveal the causal chain of events that connect variation in one quantity to variation in another. The variables and parameters in the model must be related, directly or indirectly, to quantities that biologists measure, so that experiments with the model have biological meaning. The metabolic systems that have evolved are very complicated, subtle, and difficult to understand. There is no substitute for detailed biological experimentation on the biology and the biochemistry of the parts. But mathematical models, based on the real biology, can shed light on how the parts work together and the causal relationships between them, and suggest strategies for interventions in disease states.
